# A Case Study of Photosensitivity Associated with *Anaplasma* spp. Infection in Cattle

**DOI:** 10.3390/ani12243568

**Published:** 2022-12-16

**Authors:** Alina Anton, Gheorghe Solcan

**Affiliations:** Clinics Department, Faculty of Veterinary Medicine, “Ion Ionescu de la Brad” Iasi University of Life Sciences, Mihail Sadoveanu Alley, 700490 Iasi, Romania

**Keywords:** *Anaplasma* spp., cattle, oxytetracycline, photodermatitis

## Abstract

**Simple Summary:**

A five-year-old Holstein cow in her fifth month of gestation was presented to our ruminant livestock clinic for skin injury diagnosis, which occurred after grazing with other healthy Holstein cows for several months on a grass pasture. The cow was dewormed on a regular schedule and produced a decreasing amount of milk. It was RT-PCR positive to *Anaplasma* spp. infection. After starting oxytetracycline treatment, its condition improved within 24 h, and it fully recovered within 30 days. The underlying pathogenic mechanism of *Anaplasma* spp. infection is its impairment of the bile flow and liver function, leading to secondary photosensitization due to the pathological retention of phylloerythrin. In this case, *Anaplasma* spp should be considered an etiologic diagnosis of hepatogenous photosensitization, as the cow was continually exposed to sunlight while grazing on pasture.

**Abstract:**

Anaplasma infection has not previously been reported as a cause of photosensitization. This case presents a five-year-old Holstein cow in her fifth month of gestation with skin injury of nonpigmented areas clearly delimited from adjacent unaffected pigmented skin. Specific lesions included alopecia, localized oedema, desquamation erythema, serous exudation, thick detached skin, fissures, crusting, and necrosis, indicating photodermatitis. Hematological abnormalities were leukocytosis with neutrophilia and normocytic hypochromic anemia. Based on a hemoparasitic examination of blood smears, the organism *Anaplasma* spp. was observed within the red blood cells. Biochemical analyses revealed increased serum liver enzyme activity associated with hepatocellular damage and cholestasis. This cow was RT-PCR positive for *Anaplasma* spp. infection. After treatment with oxytetracycline started, its condition improved within 24 h, and it recovered completely within 30 days. In this case, the underlying pathogenic mechanism of *Anaplasma* spp. infection facilitated the impairment of bile flow and liver function, resulting in hepatogenous photosensitization.

## 1. Introduction

Photosensitization, also known as photodermatitis, is caused by the accumulation of photosensitizing substances in the skin when exposed to light, resulting in severe dermatitis of unpigmented and unprotected skin [1]. There are three types of photosensitization: (i) Primary, in which plant toxins and photodynamic chemicals arrive at the skin through circulation; (ii) congenital porphyria, in which there is a metabolic malfunction in porphyrin metabolism; and (iii) secondary (hepatogenous), which occurs when the liver’s capacity to excrete metabolites resulting from the catabolism of dietary chlorophyll is impaired [2]. Phylloerythrin is a chlorophyll degradation product released by rumen microbial digestion and excreted in the bile. Failure to excrete phylloerythrin due to hepatic dysfunction or bile injury increases the quantity in the circulation, entering the small vessels of the skin and making it sensitive to sunlight [1]. 

Liver pathologies are the most important in Romania cattle and have been known for many years [3], especially due to rickettsiosis and anaplasmosis transmitted by ticks [4], with significant losses in local cattle. Bovine anaplasmosis is the most widespread tick-borne disease in the world and remains a serious problem with a huge economic impact [5] because of the high morbidity and mortality in the cattle population [6]. *Anaplasma marginale* is one of the most common tick-borne pathogens of cattle worldwide and is more frequently detected in Europe [7]. Transmission occurs biologically through ticks but can also occur transplacentally and mechanically via blood-contaminated objects [1].

Infection with *Anaplasma* spp. has never been previously documented as a cause of hepatogenous photosensitization in cattle. Thus, the current study describes a pregnant Holstein cow with secondary photosensitization due to the impairment of bile flow and hepatic functions caused by *Anaplasma* spp infection.

## 2. Materials and Methods

A five-year-old Holstein cow from the north-east of Romania, in her fifth month of gestation with a decrease in milk production and deworming status to date, was presented to our ruminant livestock clinic for skin injuries, which occurred after grazing for several months with thirty other healthy Holstein cattle on a grass pasture with access to mineral supplement and ad libitum water. Jugular venous blood samples were taken in tubes with EDTA for hematological examination and with a clot activator for serum biochemical analysis. Hemoglobin (Hgb), hematocrit (HCT), mean corpuscular volume (MCV), mean corpuscular hemoglobin (MCH), mean corpuscular hemoglobin concentration (MCHC), and red and white blood cells count (RBCs and WBCs, respectively) analyses were performed with an automated analyzer (VetScan HM5^®^, Abaxis^®^, Griesheim, Germany) using bovine reference levels [8]. 

The microscopic examination of a thin and thick Giemsa-stained blood smear was used to distinguish anaplasmosis from babesiosis. Serum gamma-glutamyl transferase (GGT), aspartate aminotransferase (AST), alanine aminotransferase (ALT), alkaline phosphatase (ALP), total bilirubin, creatinine, urea nitrogen, glucose, total protein, albumin, globulin, amylase, sodium, potassium, calcium, and phosphorus were measured with an automated analyzer (VetScan VS2^®^, Abaxis^®^, Griesheim, Germany) using bovine reference levels [9]. Hematological and biochemical tests were repeated thirty days later. Serology was used for the diagnosis of leptospirosis, and fecal samples were taken for parasitology tests completed by routine methods. A standard urine test strip was also completed. The real-time PCR assay (CFX96 System^®^, Bio Rad^®^, Singapore) was performed for the detection of *Anaplasma* spp., as described by Nazar et al. [10]. Skin biopsies were fixed in a 10% neutral-buffered formalin solution and processed as the standard for histopathology.

## 3. Results

**Figure 1:**
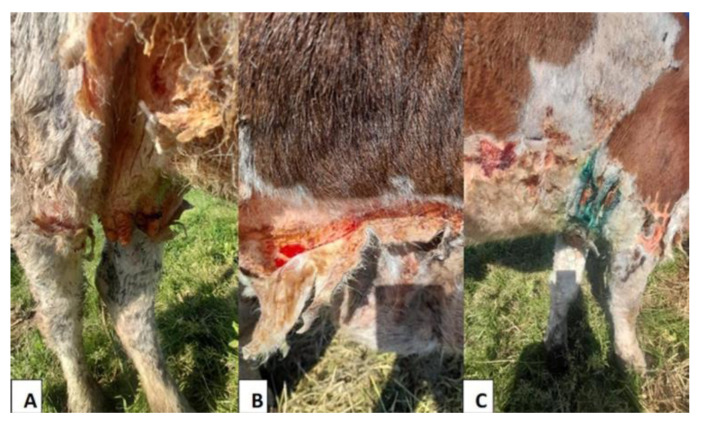
Cow with fissures and detached thick skin on the mammary gland (**A**) and the ventral area of the abdomen (**B**). Alopecia, erythema desquamation, fissures, and detached thick skin on chest and forelegs. Ointment and blue methylene applied to the lesions (**C**).

Clinical exams revealed that damaged areas of nonpigmented skin were clearly demarcated from adjacent unaffected pigmented skin. Specific cutaneous lesions included alopecia, localized oedema, desquamation erythema, serous exudation, thick detached skin, fissures, crusting, and necrosis indicating photodermatitis. Dermatitis with alopecia mainly affected the forehead, the nose, the muzzle, around the eyes, forelegs, hind legs, the ventral area of the abdomen, the chest, the back of the shoulder, teats, the perineal region, and the tail (Figure 1). The mucous membranes were pallid, and the urine was deep yellow, but the temperature, pulse, and respiration were within a normal range. No ticks were observed on this cow, but the farmer admitted that he often had problems with intensive tick infestations on his farm despite the acaricide used.

Hematological abnormalities were leukocytosis (13.61 × 10^9^/L, normal 4.9–12) with neutrophilia (8.62 × 10^9^/L, normal 1.8–6.3) and normocytic hypochromic anemia (RBCs 3.33 × 10^12^/L, normal 5.1–7.6 × 10^12^/L; Hgb 5.4 g/dL, normal 8.5–12.2; HCT 17.86 %, normal 22–33; MCHC 30.1 g/dL, normal 36–39), as presented in Table 1. A cytological examination of the blood revealed reactive lymphocytes and neutrophils, megaplatelets, and numerous platelets in the field.

**Figure 2:**
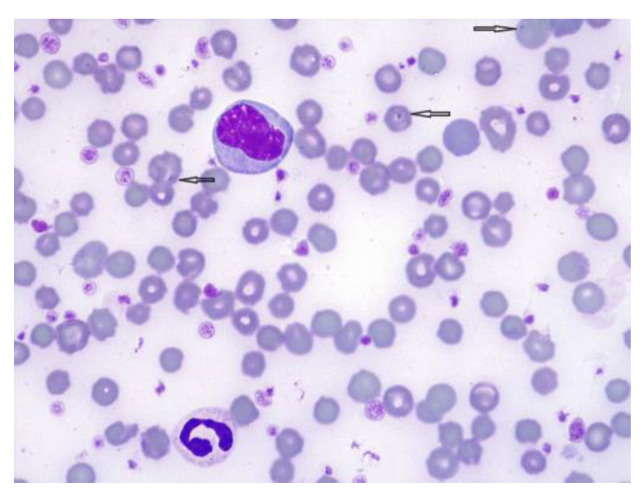
*Anaplasma* spp. on bovine blood smear, Giemsa stain, 100× oil immersion. Intraerythrocytic parasites appear basophilic and spherical and are located at the edge (*A. marginale*) or centrally (*A. centrale*).

On the basis of the hemoparasite examination of blood smears, the *Anaplasma* spp. organism within RBCs was observed (Figure 2). Microscopic examination revealed *Anaplasma* spp. as densely stained blue-violet intraerythrocytic inclusions, 0.3–1 μm in diameter. *A. marginale* was located at the edge of the erythrocyte, while *A. centrale* was more central. A cow is considered infected when one or more intraerythrocytic inclusions are observed [11].

Biochemical analyses used in this study are presented in Table 2. Albumin (1.5 g/dL, normal 3.03–3.55) and urea nitrogen (16 mg/dL, normal 20–30) from serum had values below the lower physiological limit, indicating hepatic insufficiency. Parameters such as ALT (58 IU/L, normal 11–40), AST (154 IU/L, normal 78–132), and GGT (241 IU/L, normal 15–39) were found in the serum in elevated levels, which are associated with possible hepatocellular damage and cholestasis. The total bilirubin level (0.6 mg/dL, normal 0.01–0.5) was increased according to the parameters for the species [9], associated with hepatocyte damage, cholestasis, and hemolytic anemia. Serum globulins (6.1 g/dL, normal 3–3.48) and total protein (7.6 g/dL, normal 6.74–7.46) had values that exceeded the upper physiological limit, indicating infection (namely, the superinfection of skin lesions). The serum activities of amylase, alkaline phosphatase, creatinine, glucose, phosphorus, calcium, sodium, and potassium were normal according to the parameters for the species [9].

The cow was negative for babesiosis and leptospirosis, but it was found to be positive for *Anaplasma* spp. infection. The cycle threshold value of the PCR assay for the detection of *Anaplasma* spp. was 22.92. The fecal examination for the diagnosis of parasitic infections was negative. The results from urinalysis revealed a small amount of proteinuria and bilirubinuria. 

The histopathological examination of the skin revealed diffuse parakeratotic hyperkeratosis, multifocal acantholysis, the hyperplasia of sebaceous glands, severe epidermal necrosis, the atrophy of sweat glands, the degeneration of squamous epithelial cells, and deep dermal oedema, indicating photodermatitis. The endothelial cells of dermal vessels were swollen with fibrinoid degeneration and platelet thrombosis. Microscopic examination also revealed a secondary bacterial infection.

The cow was maintained indoors during the conservative treatment, which included lanolin, aloe ointments, and blue methylene of sunlight-irritated skin tissues. Anti-inflammatory and anti-histamine injections relieved irritation and reduced self-trauma from the area affected by rubbing and kicking. After the initiation of 11 mg/kg of oxytetracycline IV (Engemycin^®^ 10%, Intervet International B.V.^®^, Boxmeer, Nederland) every 24 h for five days of treatment, the cow improved over the next 24 h and recovered completely in thirty days. One month after the initiation of treatment, hematological and biochemical analyses were normal according to the parameters for the species.

## 4. Discussion

The diagnosis of anaplasmosis is often based on the knowledge of the geographical location of the infection, typical symptoms, and abnormal laboratory findings. Endemic regions such as Romania [4] are sustained by the prevalence of both tick vectors and persistently infected cattle, which are usually asymptomatic but serve as reservoirs for disease transmission [12].

In cattle, clinical signs usually suffice for the diagnosis of photosensitization, but establishing the cause of photosensitization may be difficult [13]. A physical examination of this cow revealed lesions only on areas of nonpigmented skin. Specific cutaneous lesions ranging from alopecia to necrosis indicated photodermatitis. According to Moroni et al. [14], the absence of lesions on pigmented skin aids in the diagnosis of photosensitization. 

Photosensitization can occur hours or days after sun exposure [15] and causes damage to areas with white or thin hair. Skin necrosis, especially in white areas due to absorption and damage by ultraviolet light, occurs in all types of photosensitization [2]. When the photodynamic agent phylloerythrin accumulates in tissues, it leads to the necrosis of unpigmented skin [16]. In this case, a histopathological examination of the skin revealed lesions (parakeratotic hyperkeratosis, acantholysis, sweat glands atrophy, necrotic epidermis, and sebaceous glands hyperplasia) as reported by Minervino et al. [17] in buffalos with hepatogenous photosensitization due to liver injury caused by copper accumulation in hepatocytes. The same microscopic lesions of skin have been described by Haargis and Ginn [18] in cattle and sheep with all forms of photosensitization. Mendonça et al. [19] reported similar microscopic skin lesions in cattle, sheep, and horses with hepatogenous photosensitization caused by the ingestion of the toxic plant *Chamaecrista serpens*.

This cow had dark yellow urine but showed neither hemoglobinuria nor hematuria, which helped differentiate anaplasmosis from other hemolytic diseases. Since hemolysis occurs at the extracellular level, hemoglobinuria was not present [20]. In severely affected animals, urine often turns dark brown because of the presence of bile pigments. The mucous membranes were pale and not yellow, due to ongoing low-level hemolytic processes in which the liver excretes bilirubin at a sufficient rate to prevent jaundice.

The differential diagnoses of anaplasmosis include diseases that can cause anemia and jaundice (leptospirosis, babesiosis, and bacillary hemoglobinuria), chemicals, hepatotoxic plant poisonings, and other causes of liver disease [21].

The laboratory examination of anaplasmosis is based on a blood smear, polymerase chain reaction [22], and serological tests. Leukocytosis, neutrophilia, normocytic hypochromic anemia, and high serum liver enzymes are useful predictors of anaplasmosis. A decrease in HCT estimates the severity of the infection, especially when it drops suddenly within 24 to 48 h, reaching below 10% before death [21]. This cow had a low hematocrit, but above the severity level. 

During acute infection with *Anaplasma marginale*, 70% or more of the erythrocytes may become infected [23] causing mild to severe anemia. This is the result of the hepatic and splenic macrophage-mediated phagocytosis of infected erythrocytes [8,21]. Anemia observed in A. *marginale* infection depends on the severity of the disease. In acute forms of anaplasmosis, anemia is normocytic and progresses to macrocytic [24].

Elevated levels of ALT, AST, GGT, and total bilirubin found in this cow were also observed in infected animals with *A. marginale*, and these findings are related to the excessive destruction of erythrocytes and hepatocellular damage [25]. Hypoalbuminemia, as observed in this case, has also been observed in cattle infected with *A. marginale* as a consequence of the decreased protein synthesis capacity of the liver in chronic failure. Hepatocyte degeneration and necrosis are important characteristics of bovine anaplasmosis and are more severe than in babesiosis [26]. Acute hemolysis, such as anaplasmosis, leads to the overproduction of bilirubin and its retention in the bile ducts. Alternatively, severe liver damage prevents hepatocytes from metabolizing and excreting bile [2]. When liver function is affected, the by-products of chlorophyll digestion are not adequately excreted and too much phylloerythrin reaches the blood and travels to the skin and the animal develops photosensitization when sunshine reaches its body [27].

Plants known to have photodynamic agents as sources of primary photosensitization in cattle are Hypericum perforatum, Polygonum fagopyrum, Lolium perenne, Sphenosciadium capitellatum, Malachra fasciata, Heterophyllaea spp., Froelichia humboldtiana, and Biserrula pelecinus [28]. Regarding the secondary (hepatogenous) photosensitization, the following plants cause hepatocellular degeneration because they contain photosensitizing saponins, whose metabolites crystalize in the bile causing cholangitis and biliary obstruction in relation to Brachiaria decumbens, Nolina texana, Tribulus terrestris, Agave spp., Narthecium ossifragum, Trifolium hybridum [28], and Chamaecrista serpens [19]. None of these plants were identified on the pasture where the cow was grazing, and no cases of plant poisoning were reported by this farm veterinarian in the last five years.

The absence of serum biochemical evidence for cholestasis or liver damage is used to differentiate primary from secondary (hepatogenous) photosensitization [11]. Hepatogenous photosensitization always accompanies cholestasis lasting more than a few days in cattle that are kept in sunlight and have eaten green feed [29]. In this case, the underlying pathogenic mechanism of *Anaplasma* spp. infection is the impairment of the bile flow and leads to hepatogenous photosensitization because of pathological phylloerythrin retention when the cow is continuously exposed to sunlight. 

Less well-known causes of photosensitization following common infectious diseases have also been documented. Nieman et al. [15] raise the point that photosensitization in grazing Holstein steers does not always occur in every case of hepatic injury; it may occur in some cases of seemingly mild injury (such as hepatic lipidosis post-coccidiosis) and may not occur in the severe hepatic injury cases of other animals. Grazing conditions and factors associated with forage and chlorophyll also likely played an important role.

The most commonly used serological tests for diagnosing anaplasmosis are the indirect immunofluorescent test and ELISA, but these are not helpful for diagnostic purposes in the acute stage of the disease. The differentiation of *Anaplasma* spp. by cELISA is not possible. [30]. Studies have suggested serological cross-reactions between A. *phagocytophilum* and other *Anaplasma* spp. [31]. The same applies under certain circumstances pertaining to molecular markers [32].

This cow was found positive by the RT-PCR assay for *Anaplasma* spp. infection. This method is widely applied for the detection of pathogens [33]; it is very sensitive at first [7] but quickly loses sensitivity following appropriate antibiotic administration. A negative result does not exclude the diagnosis, as intermittent levels of bacteremia could produce false-negative results. The PCR supports diagnosis particularly in the earliest and latest stages of the disease, when the number of parasites is too low to be detected by microscopy analysis [34].

The gold standard of acute anaplasmosis diagnosis involves the identification of *A. marginale* following the microscopic examination of blood smears [6,21,35]. *Anaplasma* spp. have different cell tropism. Meanwhile, *A. marginale* and *A. centrale* are erythrocytic, *A. bovis* infect monocytes, and *A. phagocytophilum* infects granulocytes (especially neutrophils) [36].

*A. phagocytophilum* and *A. marginale* are both pathogenic in cattle and cause fever, hemolytic anemia, decreased milk production, cough and abortion, while *A. bovis* and *A. centrale* are less pathogenic and usually lead to subclinical infections [37]. A variety of erythrocyte parasites can be identified via an examination of peripheral blood smears, such as *Anaplasma* spp., *Mycoplasma* spp., *Babesia* spp., and *Theileria* spp. [38]. This cow was infected with *A. marginale* and *A. centrale* and was microscopically identified on the blood smear. 

Tetracyclines, in the form of short-acting 11 mg/kg intravenous oxytetracycline every 24 h for three to five days and long-acting 20 mg/kg intramuscular oxytetracycline at 72 h intervals, are the most successful tool in the treatment of anaplasmosis in cattle [12]. This cow received short-acting oxytetracycline for five days and fully recovered within 30 days. Supportive therapy was also important to help the animal maximize its chances of survival. 

The cattle, regardless of age, are infected with *A. marginale*, and they remain carriers of infections that are persistent for life, as characterized by recurrent cycles of rickettsemia [1,5]. During the carrier stage, these cattle with low-level *A. marginale* infections will not exhibit any associated clinical signs [39]. However, ticks feeding on blood from the recovered cattle will transmit anaplasmosis to other susceptible animals. Carriers rarely develop anaplasmosis a second time. Unidentified carriers in a herd are the likeliest source of infection for future outbreaks, but the detection of *A. marginale* in erythrocytes is unreliable because of too-low levels of parasitemia [21]. 

The control measures for anaplasmosis vary according to the epidemiological status of herds and include vaccination, tetracycline treatment, and transmission prevention by minimizing exposure to vectors through the strategic use of insecticides, pastures, and herd addition management [12].

## 5. Conclusions

This study demonstrates the involvement of external environmental factors in *Anaplasma* spp. biological infection, whereby a diet of chlorophyll-rich green feed and sunlight act together with impairment of the bile flow and liver function to induce photosensitization. 

*Anaplasma* spp. should be considered for the etiological diagnosis of hepatogenous photosensitization in cattle; however, more cases are needed to strengthen this hypothesis.

## Figures and Tables

**Table 1:** Values of hematological analysis performed in this cow.

Hematological Parameters	Values	Reference [8]
Leucocytes	13.61 *	4.9–12 × 10^9^/L
Lymphocytes	4.53	1.6–5.6 × 10^9^/L
Monocytes	0.2	0–0.8 × 10^9^/L
Neutrophils	8.62 *	1.8–6.3 × 10^9^/L
Eosinophils	0.1	0–0.9 × 10^9^/L
Basophils	0.1	0–0.3 × 10^9^/L
RBC	3.33 *	5.1–7.6 × 10^12^/L
Hemoglobin	5.4 *	8.5–12.2 g/dL
HCT	17.8 *	22–33%
MCV	50	38–50 fL
MCH	16.1	14–18 pg
MCHC	30.1 *	36–39 g/dL
Thrombocytes	641	200,000–650,000 per μL

* value outside reference limits.

**Table 2:** Values of biochemical analysis performed in this cow.

Biochemical Parameters	Values	Reference [9]
Albumin	1.5 *	3.03–3.55 g/dL
ALP	148	0–488 IU/L
ALT	58 *	11–40 IU/L
AST	154 *	78–132 IU/L
GGT	241 *	15–39 IU/L
Amylase	40	40–161 IU/L
Total bilirubin	0.6 *	0.01–0.5 mg/dL
Urea nitrogen	16 *	20–30 mg/dL
Calcium	9.7	9.7–12.4 mEq/L
Phosphorus	5.6	5.6–6.5 mEq/L
Creatinine	1.0	1.0–2.0 mg/dL
Glucose	72	45–75 mg/dL
Sodium	138	132–152 mEq/L
Potassium	4.5	3.9–5.8 mEq/L
Total protein	7.6 *	6.74–7.46 g/dL
Globulin	6.1 *	3–3.48 g/dL

* value out of reference limits.

## Data Availability

Not applicable.
